# Cognitive Impairment in Children and Adolescents With Migraine

**DOI:** 10.3389/fneur.2018.00667

**Published:** 2018-08-14

**Authors:** Cristiano Termine, Beatrice Bartoli, Massimo A. Agosti, Andrea E. Cavanna, Umberto Balottin

**Affiliations:** ^1^Child Neuropsychiatry Unit, Department of Medicine and Surgery, University of Insubria, Varese, Italy; ^2^Department of Medicine and Surgery, University of Insubria, Varese, Italy; ^3^Department of Neuropsychiatry, Birmingham and Solihull Mental Health NHS Foundation Trust and University of Birmingham, Birmingham, United Kingdom; ^4^School of Life and Health Sciences, Aston University, Birmingham, United Kingdom; ^5^Institute of Neurology, University College London, London, United Kingdom; ^6^Child Neuropsychiatry Unit, IRCCS Mondino Foundation, Pavia, Italy; ^7^Child Neuropsychiatry Unit, Department of Brain and Behavioral Sciences, University of Pavia, Pavia, Italy

**Keywords:** cognitive aspect, neuropsychology, headache, migraine, children, adolescent

## Introduction

The presence and characteristics of cognitive alterations in children and adolescents affected by migraine have been largely under-investigated. Childhood and adolescence are key periods for personal growth and academic achievements, and migraine-related cognitive deficits may interfere with functioning levels across several settings. A careful analysis of cognitive impairment in the context of migraine is pivotal for making informed decisions on the most appropriate care pathways.

## Methods

We therefore critically evaluated the results of research studies conducted to date on cognitive function in children and adolescents affected by migraine using the Pubmed database. The literature search was limited to original articles published in English language and focused on current research trends. We operationally defined cognitive processing as the range of individual cognitive functions assessed by neuropsychological studies. Our analysis, which did not include findings on cognitive processing assessed by neurophysiological measures for methodological consistency, led us to formulate the opinion that young patients affected by migraine may present with specific cognitive deficits.

## Results

An early neuropsychological study on young patients with migraine was conducted in 1989 on a group of 20 children affected by migraine without aura, aged between 7 and 11. The authors of this study did not identify clinically relevant impairment in cognitive performance, with the exception of impaired functioning in short and long-term memory tasks (1). A few years later, Haverkamp et al. (2) reported no significant differences between children with migraine aged 6–12 years and their healthy siblings on a measure of sequential and simultaneous information processing (2).

Contrarily, Riva et al. (3) reported significant alterations in the information processing rate only. Patients with migraine showed delayed reaction times to visual stimuli compared to healthy controls; interestingly, reaction times were the only parameters showing a significant correlation with the pattern of headache episodes. The authors hypothesized the existence of reduced rates of information processing speed within the posterior cortical areas involved in detecting visual stimuli and within the premotor areas responsible for programming and implementing motor responses. The findings of this study were however limited by the absence of a matched control group (3).

Villa et al. (4) conducted a neuropsychological study focusing on attentional skills in 30 young patients affected by migraine and in a control group composed by 30 healthy children. Attention is a multiform neurological function that is regulated and controlled by a wide set of anatomical structures encompassing the cerebral cortex, brainstem, and limbic system; it has also been defined as the ability to react to relevant stimuli to the detriment of others. Compared to controls, children affected by migraine exhibited an impairment in all variables with the exception of reaction times in visual attention tasks. Results showed that patients with migraine had problems in selective and alternate attention, even though the attention task achievement were within the normal range in both groups. There were no correlations between frequency or duration of migraine attacks and attention deficit were found in the migraine group (4).

Riva et al. (5) studied 62 children with different forms of primary headache compared to 52 controls. Using the Conners' Continuous Performance Test, these authors observed no significant differences in the pattern of attention between children with different forms of headache. However, results showed shorter reaction times and an increase in the number of errors in children with migraine compared to the control group; this was interpreted by the authors as suggestive of an impulsive response style in children with migraine (5).

More recently, Genizi et al. (6) retrospectively reviewed the medical records of 243 children and adolescents with primary headache and found a significantly higher prevalence of attention-deficit and hyperactivity disorder and learning disabilities (6). These findings partially overlap with the results of a previous study by Arruda and Bigal (7), which did not show any association between primary headache and attention-deficit and hyperactivity disorder (or its inattention component), whereas a significant association was found between primary headache and the hyperactive-impulsive component of attention-deficit and hyperactivity disorder (7).

A longitudinal analysis performed by Waldie et al. (8) on patients with migraine aged 3 to 26 showed deficits in verbal abilities, which appeared to be more pronounced during childhood and teenage years. Moreover, lower high school grades and examination scores reported by patients affected by migraine suggested that the subtle verbal deficits might have influenced later performance (8). In a study published in 2010, Parisi et al. reported less developed verbal abilities in school-aged children affected by headache compared to children without headache, with no discrepancy between migraine and tension-type headache. These authors also suggested that both elevated headache frequency and early age at onset could correlate with cognitive impairment, possibly due to the immaturity of the central nervous system in the developmental age. Verbal subscales appear to be more severely affected compared to performance subscales, and verbal comprehension performances appear to be more compromised than other verbal abilities. It is important to consider that linguistic abilities are needed to perform verbal tasks, in terms of both receptive and expressive functions. Moreover, language is considered a relatively recent phylogenetic acquirement and is one of the most complex abilities of humans. Language skill scores were in line with global Intelligence Quotient scores across all standardized tests (9).

In a controlled study conducted by Moutran et al. (10), 30 children and adolescents with migraine were compared to 30 control subjects. The authors found a significantly lower average Intelligence Quotient in the migraine group (102.8 vs. 113.7); both the Verbal Intelligence Quotient (102.4 vs. 113.1) and the Performance Intelligence Quotient (102.8 vs. 112.2) showed significant differences. In this study, participants were tested using the Wechsler Intelligent Scale for Children (Third Edition): the authors performed a comparison between the different factorial indexes which can be obtained by this version of the Wechsler Intelligence Scale for Children. Results showed a trend toward statistical significance in reduction of verbal comprehension (113.3 vs. 112.0) and a statistically significant reduction of perceptual organization (110.4 vs. 101.7), freedom from distractibility (110.1 vs. 98.5), and processing speed (111.6 vs. 104.2) (10).

In contrast, results from a study by Esposito et al. (11) showed differences in the cognitive profiles between patient affected by migraine without aura and those with tension-type headache; a slight decline in verbal skills and an increase in the perceptual organization abilities was detected in children affected by tension-type headache compared to children affected by migraine without aura. Patients with migraine reported reduced scores both in Full Scale Intelligence Quotient (97.31 vs. 100.23), Verbal Intelligence Quotient (102.65 vs. 105.31) and Performance Intelligence Quotient (92.73 vs. 95.02) compared to the control group (11). Parisi et al. (12) compared patients affected by Rolandic epilepsy to patients with Rolandic epilepsy and co-morbid migraine as well as patients with centro-temporal spikes and co-morbid migraine. Although there were no significant differences in terms of Intelligence Quotient, a difference was found in both migraine groups in terms of reduced long-term verbal memory using a more detailed assessment (A Developmental NEuroPSYchological Assessment—Second edition) (12).

The results of a recent study by Costa Silva et al. (13) provided confirmatory evidence that adolescents suffering from migraine may present with verbal memory and learning impairments. Patients with migraine were more significantly influenced by distractors and reported problems with recognition and recall. The poor test performance of patients with migraine suggested difficulties in registration, consolidation, and recall of verbal stimuli. These difficulties were related to changes in the ability to organize thoughts and to use scheme to search for data. The authors also found significant differences in executive functioning such as selective and divided attention, speed of processing information and visuo-motor tracking between patients with migraine and the control group (13). These results are in line with previous findings and confirm that young patients affected by migraine may present with impaired short and long-term verbal memory, speed processing information, and selective and divided attention (1, 3, 4, 8). Impairments in visuo-motor tracking and selective attention are in line with evidence on adults (14) and children (3) affected by migraine, although in a previous study on sequential and simultaneous information processing no significant differences were found between children suffering from migraine and the control group (2). Calandre et al. (14) found that the only variable showing a difference between patients with migraine and controls was reaction time. The authors hypothesized that the rate of information processing might be the first manifestation of co impairment associated with migraine and that other cognitive domains might be subsequently impaired. Problems in visuomotor processing speed are among the most commonly reported deficits in patients with white matter abnormalities. Neuroimaging studies reported non-specific findings in patients affected by migraine (14), as well as links between white matter irregularities and headache episodes in adult patients (15–17). To date, the neurochemical substrates associated to cognitive impairment in migraine are not completely known, however it has been reported that neurotransmitters such as dopamine, noradrenaline and glutamate (known to be involved with cognition) could play a central role in the pathophysiology of migraine (18).

Finally, in a referred population, students who missed more school due to headache had higher depression scores and lower academic performance than students who missed less school (19). Future studies should investigate the complex relationship between recurrent headache and school absenteeism (20).

## Discussion

In conclusion, results of research studies to date show that children and adolescents affected by migraine may present with specific cognitive deficits, such as impaired short and long-term verbal memory, speed processing information, and selective/divided attention (13). It has to be highlight that most of children and adolescent evaluated have been recruited from clinical samples and, more likely, they could have a comorbid medical condition that could influence results. Although the reviewed results are to some extent contradictory, subtle deficits in general cognitive skills may be reported by selected patient groups. As similar alterations have been described in adult patients with migraine (14, 21, 22), it remains to be clarified whether cognitive impairments persist during the entire course of life or re-emerge at later stages.

Migraine is a heterogeneous chronic disorder characterized by episodic attacks. Such heterogeneity (e.g., in the frequency of attacks, presence of aura, level of associated disability, comorbidity rate, non-headache features) can be difficult to summarize when studying associations to sensitive areas, such as cognitive changes in children. Despite our efforts to reduce methodological heterogeneity by focusing on neuropsychological studies only, the available literature shows a wide variability and it is difficult to conduct meta-analyses of association risk. The inconsistency of the findings poses considerable limitations to any conclusions about association risks, however it is possible to outline future research strategies to address unanswered questions. Specifically, future studies should be conducted on larger and more homogeneous cohorts of patients, using standardized neuropsychological batteries encompassing the broad spectrum of cognitive functions. Multicenter studies involving the coordinated research activity of specialist clinics might allow to achieve adequate standards in terms of both statistical power and inter-rater reliability. Ideally, longitudinal studies on large cohorts of patients in transition from childhood to adolescence could be conducted to assess cognitive functions and associated clinical variables across crucial developmental trajectories. Considering also that migraine is a cronic condition that tends to persist into adolescence and adulthood, it is important to recognize effective intervention that could prevent long term cognitive impairments. Finally, further studies are needed both to clarify uncertainties and to evaluate possible changes in cognitive symptoms following targeted interventions, as compared to traditional treatments for migraine (23).

## Author contributions

All authors listed have made a substantial, direct and intellectual contribution to the work, and approved it for publication.

### Conflict of interest statement

The authors declare that the research was conducted in the absence of any commercial or financial relationships that could be construed as a potential conflict of interest.
